# Loci and natural alleles underlying robust roots and adaptive domestication of upland ecotype rice in aerobic conditions

**DOI:** 10.1371/journal.pgen.1007521

**Published:** 2018-08-10

**Authors:** Yan Zhao, Hongliang Zhang, Jianlong Xu, Conghui Jiang, Zhigang Yin, Haiyan Xiong, Jianyin Xie, Xueqiang Wang, Xiaoyang Zhu, Yang Li, Weipeng Zhao, Muhammad Abdul Rehman Rashid, Jinjie Li, Wensheng Wang, Binying Fu, Guoyou Ye, Yan Guo, Zhiqiang Hu, Zhikang Li, Zichao Li

**Affiliations:** 1 Key Lab of Crop Heterosis and Utilization of Ministry of Education and Beijing Key Lab of Crop Genetic Improvement, China Agricultural University, Beijing, China; 2 Institute of Crop Science, Chinese Academy of Agricultural Sciences, Beijing, China; 3 Shenzhen Institute for Innovative Breeding, Chinese Academy of Agricultural Sciences, Shenzhen, China; 4 University of Agriculture Faisalabad, Sub-campus Burewala-Vehari, Pakistan; 5 International Rice Research Institute, Manila, Philippines; 6 State Key Laboratory of Plant Physiology and Biochemistry, College of Biological Sciences, China Agricultural University, Beijing, China; University of Minnesota, UNITED STATES

## Abstract

A robust (long and thick) root system is characteristic of upland *japonica* rice adapted to drought conditions. Using deep sequencing and large scale phenotyping data of 795 rice accessions and an integrated strategy combining results from high resolution mapping by GWAS and linkage mapping, comprehensive analyses of genomic, transcriptomic and haplotype data, we identified large numbers of QTLs affecting rice root length and thickness (RL and RT) and shortlisted relatively few candidate genes for many of the identified small-effect QTLs. Forty four and 97 QTL candidate genes for RL and RT were identified, and five of the RL QTL candidates were validated by T-DNA insertional mutation; all have diverse functions and are involved in root development. This work demonstrated a powerful strategy for highly efficient cloning of moderate- and small-effect QTLs that is difficult using the classical map-based cloning approach. Population analyses of the 795 accessions, 202 additional upland landraces, and 446 wild rice accessions based on random SNPs and SNPs within robust loci suggested that there could be much less diversity in robust-root candidate genes among upland *japonica* accessions than in other ecotypes. Further analysis of nucleotide diversity and allele frequency in the robust loci among different ecotypes and wild rice accessions showed that almost all alleles could be detected in wild rice, and pyramiding of robust-root alleles could be an important genetic characteristic of upland *japonica*. Given that geographical distribution of upland landraces, we suggest that during domestication of upland *japonica*, the strongest pyramiding of robust-root alleles makes it a unique ecotype adapted to aerobic conditions.

## Author summary

Asian cultivated rice is well-known for its rich-within-species diversity with two major subspecies, *indica* and *japonica* and subpopulation differentiation. A robust (long and thick) root system that is characteristic of upland *japonica* rice represents a predominant ecotype grown under aerobic and rain-fed conditions. In this study, we identified candidate genes for root length and root thickness, and validated five root length candidates by T-DNA insertional mutations. Further analyses of an Asian cultivated and wild rice population were performed based on random SNPs and SNPs within robust loci. The findings hold promise for application in improving drought resistance and also reveal the adaptive domestication history of upland rice as a unique Asian cultivated rice ecotype.

## Introduction

Asian cultivated rice (*Oryza sativa* L., *O*. *sativa*) is a staple food for half the world’s population. Grown in diverse environments worldwide, *O*. *sativa* is also well-known for its rich-within-species diversity with two major subspecies, *indica* (also referred to as *Xian*) and *japonica* (also referred to as *Geng*) and subpopulation differentiation [1–6]. Upland rice, particularly upland *japonica* rice, represents a predominant ecotype grown under aerobic and rain-fed conditions in mountainous areas of Southwest China, South and Southeast Asia, Africa and Latin America [7–11]. Most upland rice accessions have a robust root (long and thick) system [10,12,13] that confers the ability of upland to adapt to aerobic and drought-prone conditions of rain-fed, hilly environments by absorbing more water and nutrients from deeper soil zones [14–18], implying that robust roots could be a key physiological feature selected during adaptation to aerobic conditions of upland rice. Thus, exploration of loci and natural alleles underlying robust roots and adaptation analysis of upland rice using these loci are conducive to developing new rice varieties with improved drought resistance and water use efficiency, when plant breeders are facing an increasing challenge of water shortage caused by global climate change.

Breeding rice lines with robust roots based on phenotypic selection has been challenging because conventional breeding approaches based on phenotypic selection are ineffective for improving root traits [19–21]. Thus, tremendous efforts have been made in identifying QTLs/genes contributing to robust roots to facilitate marker- or genome-assisted breeding of drought resistance rice varieties by improving root traits. To date, many QTLs for root length (or depth) and thickness in rice have been identified in bi-parental crosses, such as *DRO1*, *DRO2*, *DRO3*, *SOR1*, *qSOR1*, *qRL6*.*1*, *qRL7* and *qRT9* [22–30]. *OsbHLH120* that controls root thickness was cloned by map-based cloning using introgression lines [23,31]. Additionally, *DRO1* affects deep rooting by controlling root angle and the mutation of *SOR1* leads to observed deficiency in root gravitropic response and soil-surface rooting [30,32]. Traditional methods using linkage mapping and map-based cloning for QTL identification and cloning are laborious and inefficient. Thus, many questions remain to be answered in understanding allelic diversity at QTL controlling robust roots in the primary gene pool of rice and their roles in the adaptation to aerobic conditions of upland rice.

Genome-wide association and transcriptome analyses have become feasible with recent advances in high-throughput sequencing technologies, which enable capture of genomic variation affecting complex traits. These include genome-wide association study (GWAS) [33–36], genome-wide screening of elite single-nucleotide polymorphism (SNP) alleles [12], determination of genome-wide expression profiles under different drought stress conditions [37–39], MutMap based on whole-genome sequencing of bulked DNA of F_2_ segregants [40], Ho-LAMap that joins GWAS and multiple bi-parental linkage analysis [41]. Using these methods, wide natural variation was explored at loci associated with root architecture, such as *Nal1*, *OsJAZ1* and *Nced* [12,36]. Although each of these approaches has advantages and disadvantages in revealing specific aspects of the genetic and/or molecular mechanisms underlying complex traits, when taken together, they provide a powerful strategy for genetic and molecular dissection of complex traits.

Based on hydrological conditions ranging from fully aerobic, temporarily and fully anaerobic, four major rice ecosystems were categorized by International Rice Research Institute (IRRI), including upland, rainfed lowland, irrigated (also called lowland or paddy) and flood-prone (also called deepwater) [7,33,42,43]. From the 1970s many morphologic and genetic studies distinguished upland and lowland rice genotypes, and investigated the domestication of upland rice [4,13,18,44]. Among morphological traits for drought resistance, such as root characteristics [45], leaf rolling [46] and ratio of shoot and root [47], root length and root thickness have been positively related to field drought resistance [48,49], implying that robust roots can be used to distinguish upland and lowland rice. Further genetic differentiation between upland and lowland rice was explored by simple sequence repeats (SSRs) located in expressed sequence tags (ESTs) isolated from rice in response to drought treatment [44,50]. These previous studies suggested that there was considerable morphologic and genetic differentiation between upland and lowland rice. Recently, a phylogenetic analysis of 3,029,822 SNPs detected from 166 rice accessions (82 lowland accessions, 84 upland accessions and 25 wild rice accessions) showed that all upland *japonica* accessions clustered together, suggesting that upland *japonica* has a single origin [18]. Despite these advances, deep sequencing of a wider collection of *O*. *sativa* was needed to investigate the adaptation to aerobic conditions of upland rice. Moreover, given differences in phylogenetic trees using whole-genome sequencing data and domestication loci [2], genetic analysis using loci associated with robust roots may be a better way to gain insights into the adaptive domestication process of upland rice.

In this study, we demonstrate an integrated strategy that combined high resolution mapping by GWAS and linkage mapping, comprehensive bioinformatics analyses of genomic, transcriptomic and haplotype data from deep genomic and transcriptomic sequencing, and large scale phenotyping of two large sets of *O*. *sativa* accessions for genetic dissection and high efficiency cloning of small-effect QTLs underlying robust roots in rice. Our results revealed insights into the adaptive domestication history of upland rice as a unique *O*. *sativa* ecotype that is adapted to the aerobic conditions of highlands in tropical and subtropical environments.

## Results

### Population structure and phenotypic characterization of root traits of 795 *O*. *sativa* accessions

**Fig 1:**
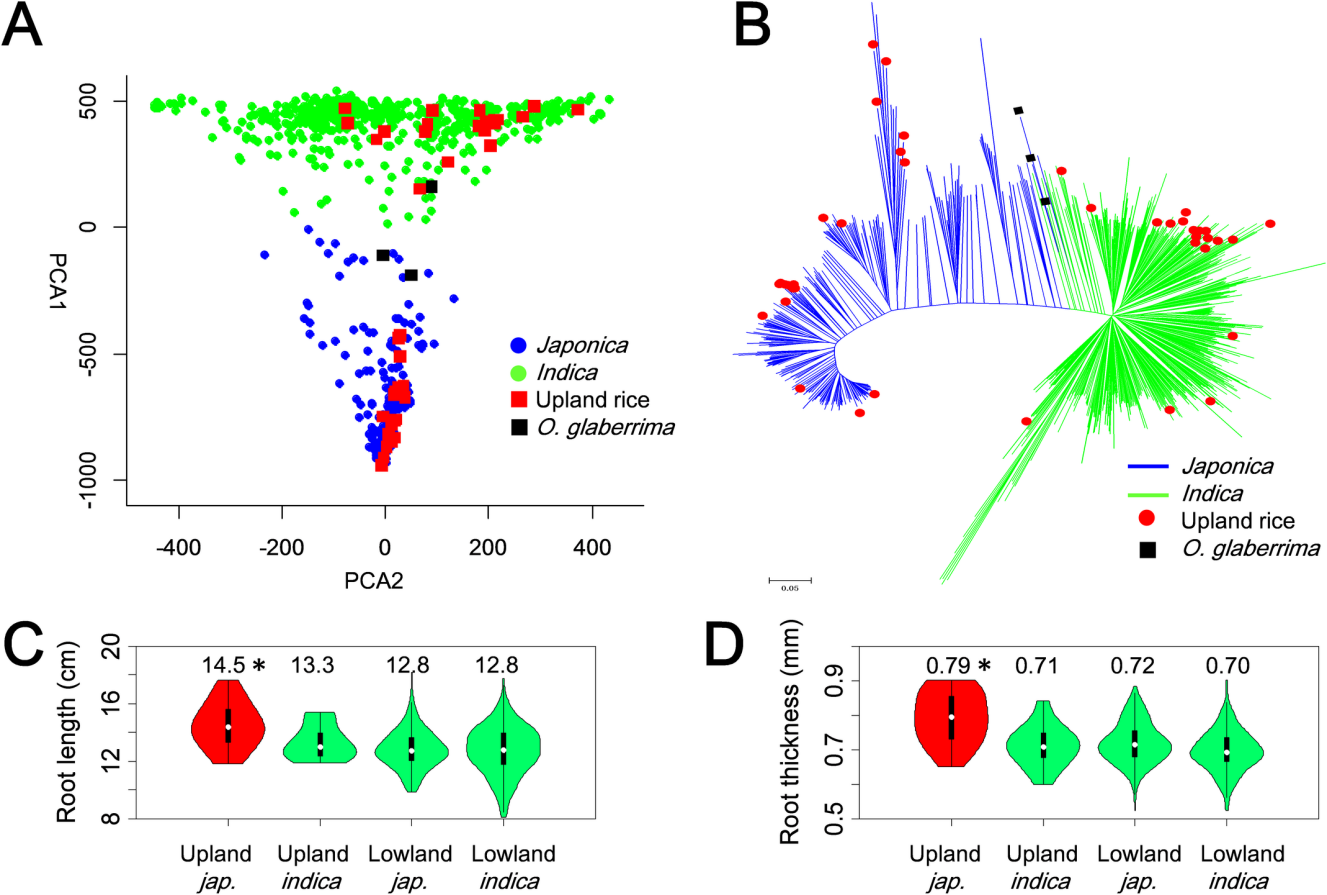
Population structure of 795 *O*. *sativa* (rice) accessions, phenotypic diversity in root traits. (*A*) Principal component (PC) plots of different ecotypes and subpopulations; and (*B*) neighbor-joining tree of all accessions and distribution of upland rice accessions in the N-J tree. PC analysis was performed using 3.3 million SNPs with missing data rates ≤ 50% and minor allele frequency ≥ 5%. N-J tree was constructed using 54,853 SNPs evenly distributed throughout the genome. Distribution of (*C*) root length and (*D*) root thickness among different rice ecotypes. Y-axes show the median (white point), 95% of confidence intervals (black bars) and range (colored shapes) of the trait phenotypes. X-axes indicate different ecotypes, ordered by upland *japonica*, upland *indica*, lowland *japonica* and lowland *indica*. Numbers above violins are mean phenotypic values of different populations. Ecotypes colored red with * were significantly higher than those colored green (*P* < 0.05 detected by one-way ANOVA).

The 795 *O*. *sativa* accessions used were from 41 countries worldwide, including a 189 accession mini-core collection retaining 70.65% of the genotypic variation and 76.97% of the phenotypic variation of 4,310 Chinese *O*. *sativa* accessions [51], and 525 lines in the International Rice Molecular Breeding Network [52], including 40 upland accessions (**S1 Table**). By comparison with the RGAP 7 Nipponbare reference genome, we obtained 15,133,187 SNPs from the 795 genomes with an average sequencing depth of 15×, including 12,648,777 SNPs with missing rates ≤ 50% **(S1A Fig)**. After removing SNPs with missing rates > 50% and minor allele frequencies < 5%, we identified 3,291,151 (3.3 million) SNPs representing high density. Using 154,516 SNPs with missing rates ≤ 50%, minor allele frequencies ≥ 5% and *r*^2^ of linkage disequilibrium (LD) ≤ 0.3, we calculated varying levels of *K* means, the *Indica* and *Japonica* varietal groups appeared clearly at *K* = 2 **(S1B and S1C Fig)**, which was supported by principal component (PC) plotting, and a kinship matrix constructed using 3.3 million SNPs and neighbor-joining tree constructed using 54,853 evenly distributed SNPs **(Fig 1A and 1B, S1D Fig)**. Referring to the recent research results of 3,010 rice accessions [1], we classified the 795 *O*. *sativa* accessions into two major subspecies, *indica* (506 accessions) and *japonica* (289 accessions) for further phenotypic analysis and GWAS in two subpopulations **(Fig 1A and 1B, S1 Table)**.

The 795 *O*. *sativa* accessions showed significant amount of variations for the measured root traits, ranging from 8.1 to 19.4 cm for root length (RL) and from 0.46 to 0.95 mm for root thickness (RT) **(S2 Fig, S2 Table)**. Upland *japonica* accessions had mean RL and RT of 14.5±1.6 cm and 0.79±0.07 mm, significantly higher than those of the other three ecotypes (upland *indica*, lowland *indica* and lowland *japonica*) **(Fig 1C and 1D)**. No obvious differences in mean root weight were detected between the upland *japonica* and other ecotypes, although there was considerable variation in root weight among different accessions within each subpopulation **(S3 Fig, S2 Table)**. Clearly, upland *japonica* accessions as a group showed extreme phenotypes for robust root systems, and our population showed sufficient variation in RL and RT to uncover the genetic architecture of robust roots.

### Identification of robust root QTLs by GWAS

**Fig 2:**
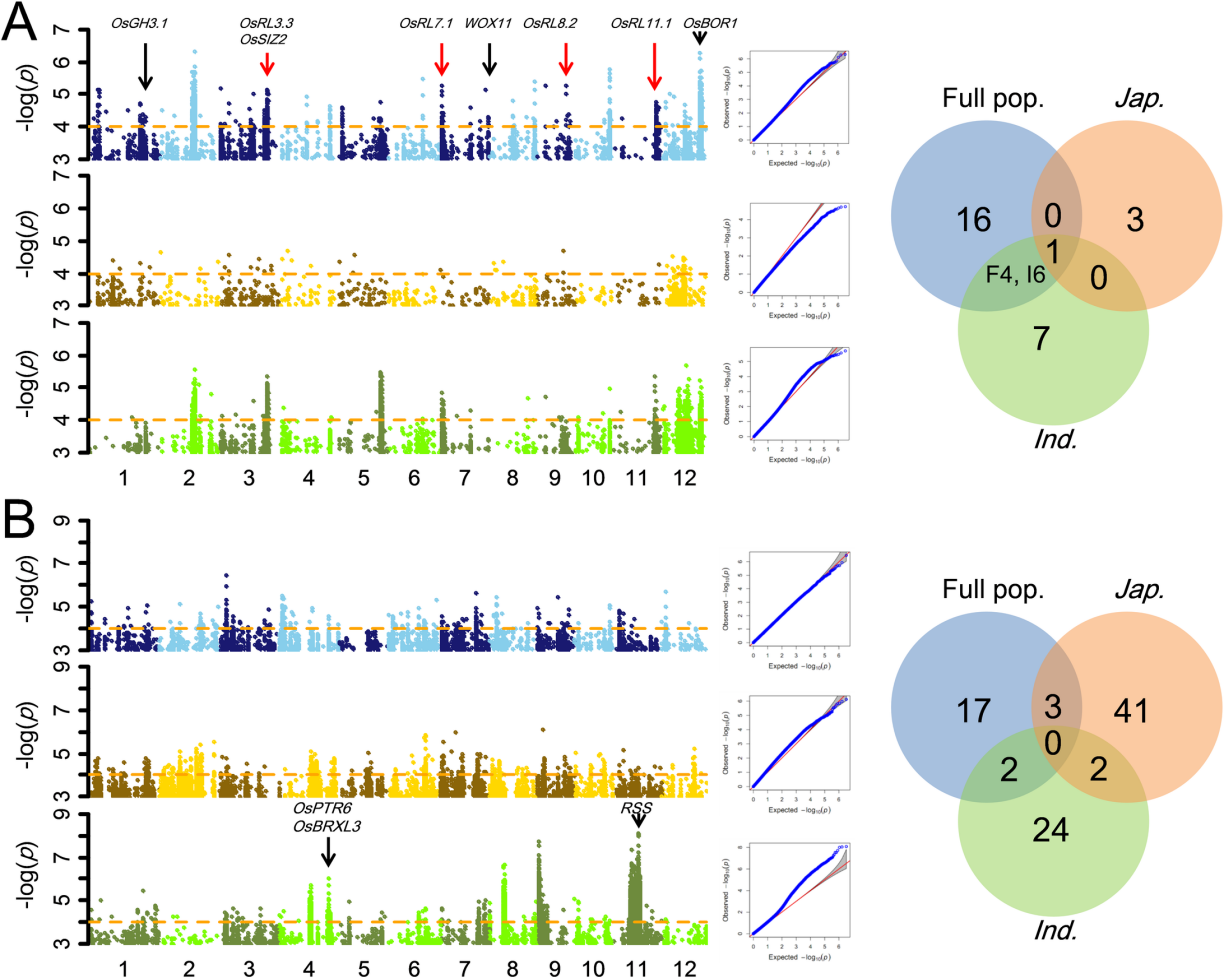
Identification of robust-root QTLs by GWAS. Manhattan plots and quantile-quantile (Q-Q) plots in the whole population (upper left, blue), *japonica* (middle left, yellow) and *indica* (lower left, green) and Venn diagram of QTL number detected in different populations (right) for (*A*) root length and (*B*) root thickness. Black and red arrows mark six known genes and five QTL genes identified/confirmed in this study in their corresponding QTLs, respectively. F4 and I6 in the Venn diagram showed that 4 QTLs in whole population overlapped with 6 QTLs in *indica*.

Using the root trait data and 3.3 million SNPs, we performed GWAS to identify important QTLs for RL and RT. To minimize false positives due to population structure [35,53,54], we compared the general linear model (GLM) **(S4 and S5 Figs)** and compressed mixed linear model (CMLM) **(Fig 2)** and determined that the CMLM model effectively minimized false positive rates in detecting RL and RT QTLs in our populations. A threshold of -log(*P*) = 4 was determined using a conditional permutation test (**S6 Fig**). We defined a QTL as including ≥ 3 clustered significant SNPs within distances ≤ 170 kb between adjacent ones, given LD decay values of ~123 kb and ~167 kb in *indica* and *japonica* populations, respectively [53]. With this criterion we identified 21, 14 and 4 RL QTLs in the whole, *indica* and *japonica* populations, respectively **(Fig 2)**. Detailed comparisons indicated that 1 of 4 QTLs in *japonica* and 7 of 14 QTLs in *indica* mapped to identical positions as RL QTLs identified in the whole population, while most other QTLs detected in both subpopulations were near those identified in the whole population, indicating that the slight differences in position of detected RL QTLs resulted from LD and population structure **(Fig 2, S3 Table)**. We also identified 22, 28 and 46 RT QTLs in the whole, *indica* and *japonica* populations **(Fig 2, S4 Table)**. Given that most of RL QTLs in both subpopulations could be identified in the whole population, the 21 RL QTLs in the whole population and 96 RT QTLs in all three populations were considered target QTLs for further exploration of functional genes.

### Determination of candidate genes within the RL and RT QTLs

We took a four-step integrated strategy to determine the high-confidence candidate genes for the detected RL and RT QTLs (see Materials and Methods for detail).

**Fig 3:**
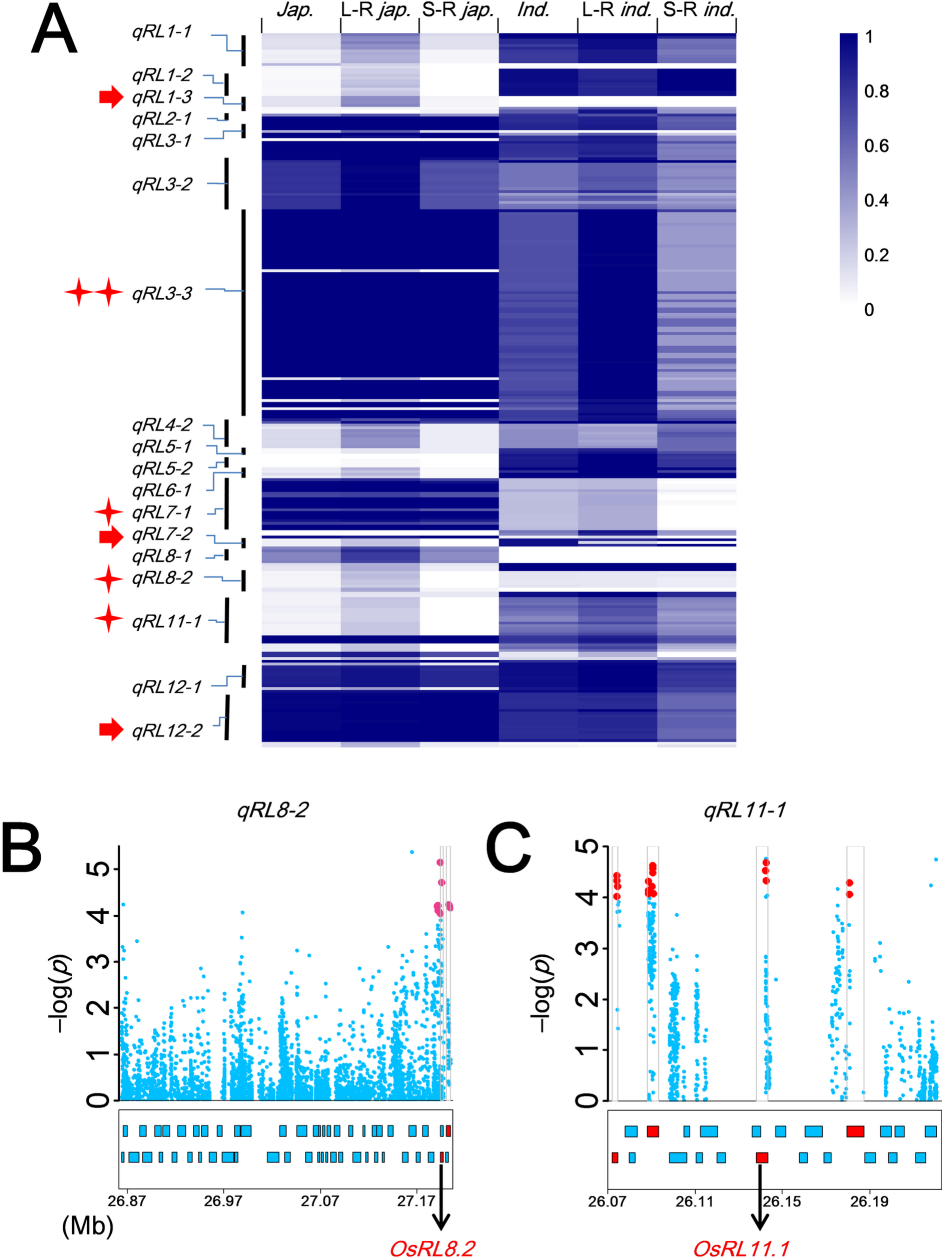
Determination of candidate genes for root-length QTLs by integrated analyses of GWAS, linkage mapping and pooled-χ2-tests. (*A*) Heat map showing significant differences in long-root allele frequencies at 230 SNPs in 85 genes in 18 GWAS detected root-length QTL regions of the two extreme phenotypic pools (long-root vs short-root varietal pools) within rice populations (**S1 Table**), in which different colors show different long-root allele frequencies; rows of the heat map are SNP sites and QTLs; columns are long-root allele frequencies in different populations (*japonica*, long-root *japonica*, short-root *japonica*, *indica*, long-root *indica* and short-root *indica*); red arrows and stars show QTLs with known genes and candidate genes identified and confirmed for root length in this study. Identification of candidate genes in regions of (*B*) *qRL8-2* and (*C*) *qRL11-1*. The top of each panel shows the entire QTL region identified by GWAS using the whole population and CMLM, and X-axis indicates the physical position (Mb) of the QTL regions in the rice genome. Negative log10-transformed *P* values are plotted on the vertical axis, in which dots show positions and -log(*P*) values of all significant SNPs in the QTL region and the red points indicate SNPs with significant differences in allele frequency of GWAS detected QTLs between the root-length pools and differential alleles between parents in previously reported bi-parental mapping populations; pink points represent SNPs with significant differences in allele frequency between pools, when the QTL was mapped only in our GWAS; and skyblue points show others. At the bottom of each panel, annotated genes are indicated by skyblue boxes; candidate genes are colored in red. Functional genes verified are red-labeled.

First, we constructed two varietal pools for RL and RT from each of the *japonica* and *indica* subpopulations with each pool containing 20 accessions with extreme phenotypes, i.e. a long-root pool and a short-root pool for RL, or a thick-root pool and a thin-root pool for RT **(S1 Table)**. We then performed pooled-χ^2^-tests to compare the allele frequencies of all significant SNPs at the target QTLs between the two pools for each root trait because varieties with extreme phenotypes were expected to possess more QTL alleles for increased trait value, whereas those of extremely low phenotypic values would have fewer. Within the 21 RL QTLs, there were 254 annotated genes containing 857 significant SNPs **(S3 Table)**. Among them, there were 174 genes with 282 large-effect SNPs in the open reading frames (ORFs) and 141 genes with 271 significant SNPs in their 2000 bp upstream promoter regions. The pooled-χ^2^-tests based on allelic frequency differences for SNPs around each detected QTL between the long-root and short-root pools allowed us to reduce the number of significant SNPs (candidate genes) from 857 to 230 (from 254 to 85) for 18 RL QTLs with QTL alleles for increased root length were much more abundant in the long-root varieties than in the short-root varieties at all 230 SNPs **(Fig 3A, S5 and S6 Tables)**.

We then compared the QTLs identified by GWAS with QTLs detected previously by linkage mapping in two bi-parental crosses and found 10 [55–60] of the 21 RL QTLs and 23 [22,23,25,59,61] of the 96 RT QTLs were common **(S7 Table)**. We screened all 380 SNPs significantly associated with RL in 10 common QTL regions containing 102 candidate genes and found that 119 of these SNPs located in 35 genes in 7 QTLs showed differences between the parents of the bi-parental populations **(S8 Table)**. Using the results of pooled-χ^2^-tests, we shortlisted the candidate genes from 254 to 81 within 17 RL QTLs **(Fig 3B and 3C, S7 Fig)**. There were 146 significant SNPs in the 81 genes, including 82 SNPs in ORFs of 42 candidate genes, while 77 SNPs in the promoters of 46 candidate genes **(S9 Table)**. These candidate genes included *OsGH3*.*1*, *WOX11* and *OsBOR1* with known functions in root development. Similar analyses were performed on the 96 RT QTLs and resulted in 466 genes as the most likely candidates for the 29 RT QTLs **(S10 Table)**.

**Fig 4:**
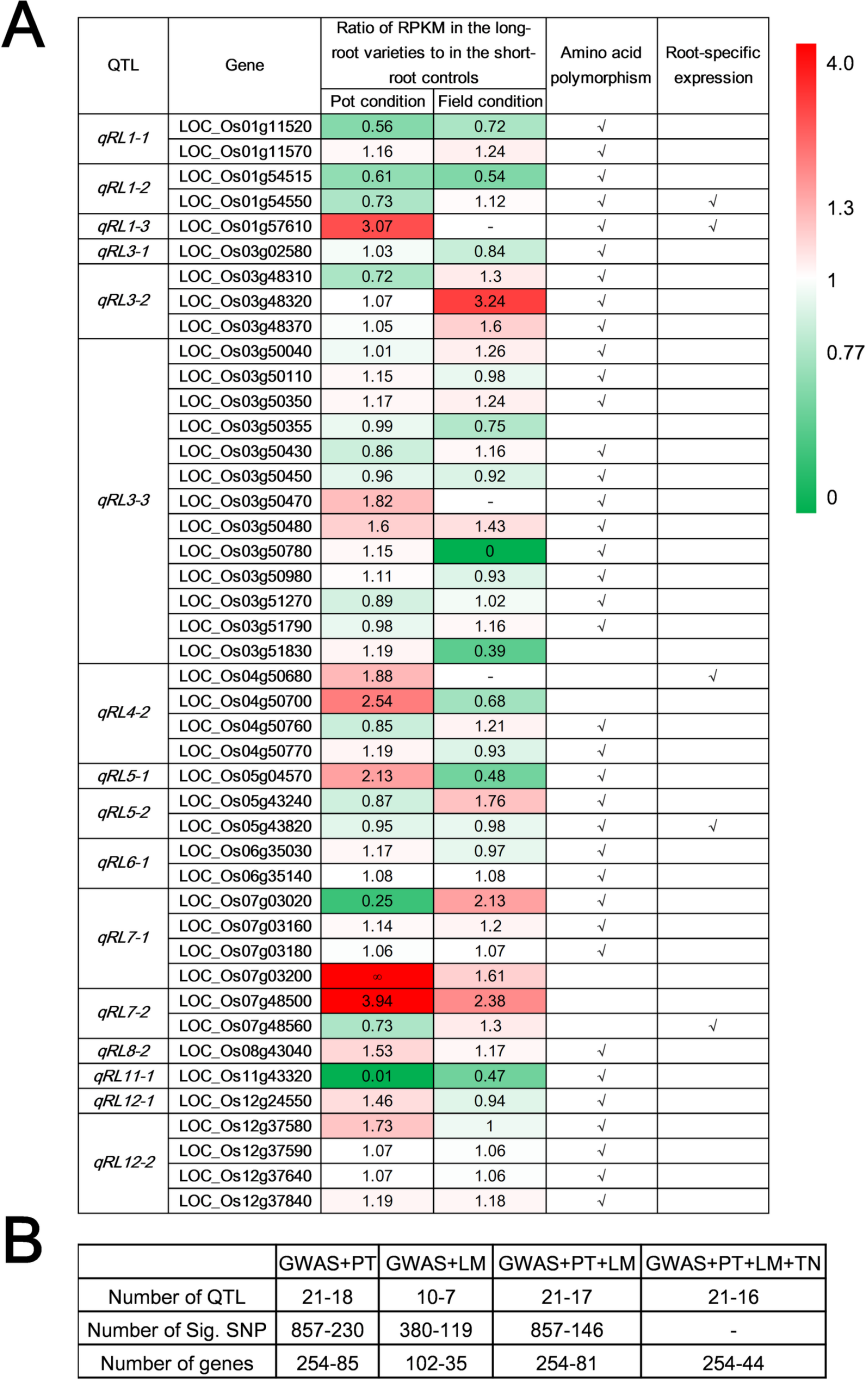
Determination of candidate genes for root-length QTLs by integrated genomic and transcriptomic analyses. (*A*) Heat map of the RPKM ratio in long-root varieties to short-root controls. Different colors show ratios and blanks indicate no expression. Rows of the heat map correspond to the 44 candidate genes for root length QTLs listed on the left of the table. The columns indicate the phenotypic differences between the long-root and short-root pools in the pot and field conditions. √ or × in column ‘Amino acid polymorphism’ indicates the presence or absence of large-effect SNPs/InDels and the significant phenotypic differences between the varietal pools of extreme phenotypes, parents or populations; column ‘Root-specific’ shows the root-specific expression of the listed genes. Detailed expression and large-effect SNPs/InDels with amino acid polymorphisms are listed in **S11–S13 Tables.** (*B*) Screening effectiveness of each step in the integrated strategy. ‘PT’, ‘LM’ and ‘DE’ indicate ‘pooled-χ^2^-tests’, ‘linkage mapping’ and ‘transcriptomic and non-synonymous SNPs analysis’. Numbers in the block represent the number of QTLs, significant SNPs and candidate genes before and after screening.

In steps 3 and 4, to further reduce the number of candidate genes for the identified root trait QTLs, we performed transcriptomic analyses on 6 robust-root *japonica* upland varieties and 6 non-robust-root lowland *japonica* lines. We expected that most real QTL genes in these two pools of varieties might indicate differences in three patterns: root-specific expression, differential expression between two pools, and no differential expression but having significant phenotypic differences between alleles at non-synonymous SNP. Based on thresholds of (RPKM, Reads Per Kilobase per Million mapped reads) RPKM_Robust-roots_/RPKM_Controls_ > 1.3 or < 0.77 between the robust-root varieties and non-robust controls in our transcriptomic data, we identified 44 candidate genes for 16 RL QTLs, including 23 genes showing root-specific differential expression between the long-root and short-root pools, 37 genes containing many large-effect non-synonymous SNPs or InDels between the long-root and short-root groups, and 5 specifically expressed root genes **(Fig 4A, S11–S13 Tables)**. Applying the same strategy, 97 candidate genes were shortlisted for 20 of the RT QTLs **(S14 and S15 Tables)**. Of all these candidate genes, six (*OsGH3*.*1* for *qRL1-3*, *WOX11* for *qRL7-2*, *OsBOR1* for *qRL12-2*, *OsPTR6* for *qRT4-4*, *OsBRXL3* for *qRT4-4*, and *RSS3* for *qRT11-4*) appeared to be the most likely QTL candidate genes based on the combined genome mapping and transcriptomic evidence **(Figs 2 and 4B)**. *OsGH3*.*1* (LOC_Os01g57610) is an indole-3-acetic acid (IAA) amido synthetase, a member of the GH3 family in which several genes are known to control root architecture and drought resistance [62–64]. *WOX11* (LOC_Os07g48560) was reported to be a key regulator specifically expressed in rice crown roots [65,66]. *OsBOR1* (LOC_Os12g37840) was reported to regulate root development in rice grown under limited boron availability [67]. Of the three genes for RT, over-expression of *OsPTR6* (LOC_Os04g50950) increases root growth in rice [68], whereas *OsBRXL3* (LOC_Os04g51172) is a member of the BREVIS RADIX family, which is known to influence cell proliferation and root elongation in Arabidopsis and rice [69]. *RSS3* (LOC_Os11g25920) regulates root cell elongation under salinity stress [70].

### Identification of important QTL alleles and validation of the QTL candidate genes

**Fig 5:**
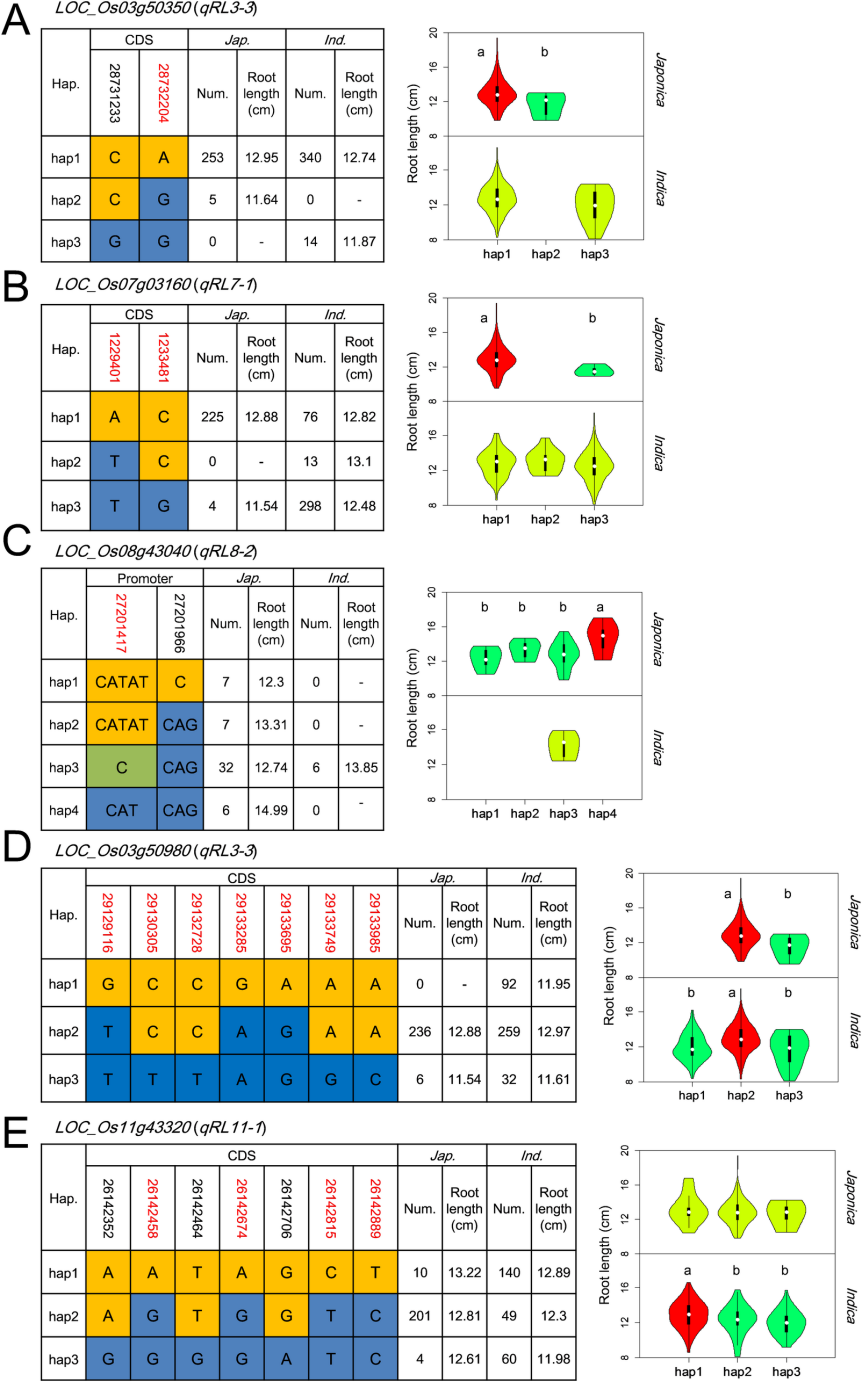
Haplotype analyses of five QTL genes for root length. Gene structures (left) and root lengths of different haplotypes (right) of (*A*) *OsRL3*.*3* (LOC_Os03g50350) for *qRL3-3*, (*B*) *OsSIZ2* (LOC_Os03g50980) for *qRL3-3*, (*C*) *OsRL7*.*1* (LOC_Os07g03160) for *qRL7-1*, (*D*) *OsRL11*.*1* (LOC_Os11g43320) for *qRL11-1*, (*E*) *OsRL8*.*2* (LOC_Os08g43040) for *qRL8-2*, where red colored numbers show the key positions where amino acid substitutions occurred among major haplotypes, that resulted in significant differences in root length; among violin maps of different haplotypes in subpopulations *japonica* (upper right) and *indica* (lower right), different letters above the violins indicate significant differences (*P* < 0.05) when analyzed by Duncan’s test or independent-sample t-tests.

**Fig 5, S8 and S9 Figs** show results from haplotype analyses using all non-synonymous SNPs and InDels within the ORFs and promoters of all candidate genes for the identified RL and RT QTLs. Significant phenotypic differences were detected between or among different haplotypes at 9 loci for 7 RL QTLs and 14 candidate genes for 7 RT QTLs with 2–5 major haplotypes at each of them. Gene ontology analysis indicated that 16 of the 23 genes have diverse biological functions and the remaining 7 genes have no known functions **(Table 1)**.

**Table 1:** Description of 9 and 14 genes for root length and root thickness identified by an integrated analyses.

Trait	QTL	Name	ID.	Number of haplotypes	Number of non-synonymous SNP and key InDel	Subpopulations with superior haplotypes	π_non-L-T-root_ / π_L-T-root_ in *japonica*	π_non-L-T-root_ / π_L-T-root_ in *indica*	π_w_ / π_J_	π_w_ / π_I_	Description
RL	*qMRL1-1*	*OsRL1*.*1*	LOC_Os01g11520	4	2	All	0.88	1.27	0.75	1.25	RING-H2 finger protein, putative, expressed
	*qMRL1-3*	*OsGH3*.*1*	LOC_Os01g57610	4	3	*Japonica*	3.71	1.03	1.04	0.83	*OsGH3*.*1*—Probable indole-3-acetic acid-amido synthetase, expressed
	*qMRL3-2*	*OsRL3*.*1*	LOC_Os03g48370	4	3	All	15.92	1.51	3.86	0.79	Disease resistance RPP13-like protein 1, putative, expressed
	*qMRL3-3*	*OsRL3*.3*	LOC_Os03g50350	3	2	All	102.07	2.84	16.96	2.56	Coatomer alpha subunit, putative, expressed
		OsSIZ2*	LOC_Os03g50980	3	7	All	29.64	1.12	5.18	1.05	ATSIZ1/SIZ1, putative, expressed
		*OsRL3*.*2*	LOC_Os03g50470	2	2	All	15.02	2.24	5.79	1.01	Expressed protein
	*qMRL7-1*	*OsRL7*.1*	LOC_Os07g03160	3	2	All	3.46	1.93	10.95	2.82	WD domain, G-beta repeat domain containing protein, expressed
	*qMRL8-2*	*OsRL8*.2*	LOC_Os08g43040	4	2	Upland *japonica*	1.69	1.23	1.79	2.10	Transferase family protein, putative, expressed
	*qMRL11-1*	*OsRL11*.1*	LOC_Os11g43320	3	7	All	1.05	0.85	0.89	0.54	NBS-LRR type disease resistance protein, putative, expressed
RT	*qBRT2-2* (J)	*OsRT2*.*1*	LOC_Os02g13400	3	4	*Japonica*	0.59	2.39	0.88	4.81	Expressed protein
	*qBRT6-2* (J)	*OsRT6*.*1*	LOC_Os06g36810	2	2	All	1.48	0.80	1.70	0.96	Expressed protein
	*qBRT6-3* (J)	*OsRT6*.*2*	LOC_Os06g37840	5	5	*Japonica*	1.53	1.27	0.86	0.95	Resistance protein, putative, expressed
	*qBRT4-1* (F)	*OsRT4*.*1*	LOC_Os04g02780	3	2	All	1.41	0.99	0.67	0.80	Amidase family protein, putative, expressed
	*qBRT4-1* (I)	*OsRT4*.*2*	LOC_Os04g30490	3	2	All	0.61	2.50	0.89	1.04	MATE efflux family protein, putative, expressed
		*OsRT4*.*3*	LOC_Os04g30920	3	10	*Indica*	12.85	4.43	3.11	0.53	Expressed protein
		*OsRT4*.*4*	LOC_Os04g31000	3	6	All	3.65	4.65	2.04	0.68	Methyltransferase domain containing protein, expressed
		*OsRT4*.*5*	LOC_Os04g31010	3	2	*Indica*	10.85	3.65	4.35	0.81	Expressed protein
		*OsRT4*.*6*	LOC_Os04g31020	3	7	*Indica*	2.80	3.34	3.29	0.91	Expressed protein
		*OsRT4*.*7*	LOC_Os04g31120	3	5	*Indica*	5.60	2.30	4.36	0.83	*OsFBK14*—F-box domain and kelch repeat containing protein, expressed
	*qBRT4-4* (I)	*OsRT4*.*8*	LOC_Os04g51060	3	9	*Indica*	2.93	2.25	3.31	0.60	Expressed protein
		*OsRT4*.*9*	LOC_Os04g51080	2	2	*Indica*	1.87	1.66	2.14	0.67	Scramblase, putative, expressed
	*qBRT8-2* (I)	*OsRT8*.*1*	LOC_Os08g14660	5	12	All	1.12	0.99	1.34	0.87	SET domain containing protein, expressed
		*OsRT8*.*2*	LOC_Os08g15050	3	2	*Indica*	0.64	1.46	0.91	0.97	CCT/B-box zinc finger protein, putative, expressed

*, Root length candidate gene is validated by T-DNA insertional mutation.

"F", "J" and "I" in bracket showed that the GWAS QTL was detected in whole population, *japonica* and *indica*, respectively.

For RL candidate genes, we detected 3 haplotypes at LOC_Os03g50980 (*OsSIZ2*) with Hap1 present only in some *indica* accessions. Hap2 was the predominant one and was associated with longer RL in both *indica* and *japonica* populations, while Hap3 had a low frequency and was associated with shorter roots in both populations **(Fig 5D)**. Similarly, three major haplotypes were detected at LOC_Os11g43320 (*OsRL11*.*1*) with Hap1 associated with longer roots. Its frequency was high in *indica* and low in *japonica* **(Fig 5E)**. The remaining 7 RL loci showed greater *indica*-*japonica* differentiation, with 1–3 of the haplotypes absent in either *indica* or *japonica* accessions **(Fig 5A–5C, S8 Fig)**. There were *japonica*-specific allele variations for RT on LOC_Os01g57610 (*OsGH3*.*1*) and LOC_Os08g43040 (*OsRL8*.*2*) since their haplotype differences were present only in *japonica* accessions, whereas LOC_Os03g50470 (*OsRL3*.*2*) showed *indica*-specific allele variations for RT.

We detected 2 to 5 haplotypes and observed a similar *indica*-*japonica* differentiation at the 14 candidate loci for RT QTLs **(S9 Fig)**. Of these, LOC_Os02g13400 (*OsRT2*.*1*), LOC_Os04g02780 (*OsRT4*.*1*), LOC_Os04g31000 (*OsRT4*.*4*), and LOC_Os06g37840 (*OsRT6*.*2*) showed *japonica*-specific allele variations for RT since significant phenotypic differences were present only between or among *japonica* haplotypes. Similarly, LOC_Os04g30920 (*OsRT4*.*3*), LOC_Os04g31010 (*OsRT4*.*5*), LOC_Os04g31020 (*OsRT4*.*6*), LOC_Os04g31120 (*OsRT4*.*7*), LOC_Os04g51060 (*OsRT4*.*8*), LOC_Os04g51080 (*OsRT4*.*9*), and LOC_Os08g15050 (*OsRT8*.*2*) possessed *indica*-specific allele variation. Only three loci, LOC_Os04g30490 (*OsRT4*.*2*), LOC_Os06g36810 (*OsRT6*.*2*), LOC_Os08g14660 (*OsRT8*.*1*) showed significant phenotypic differences between haplotypes in both *indica* and *japonica* populations.

**Fig 6:**
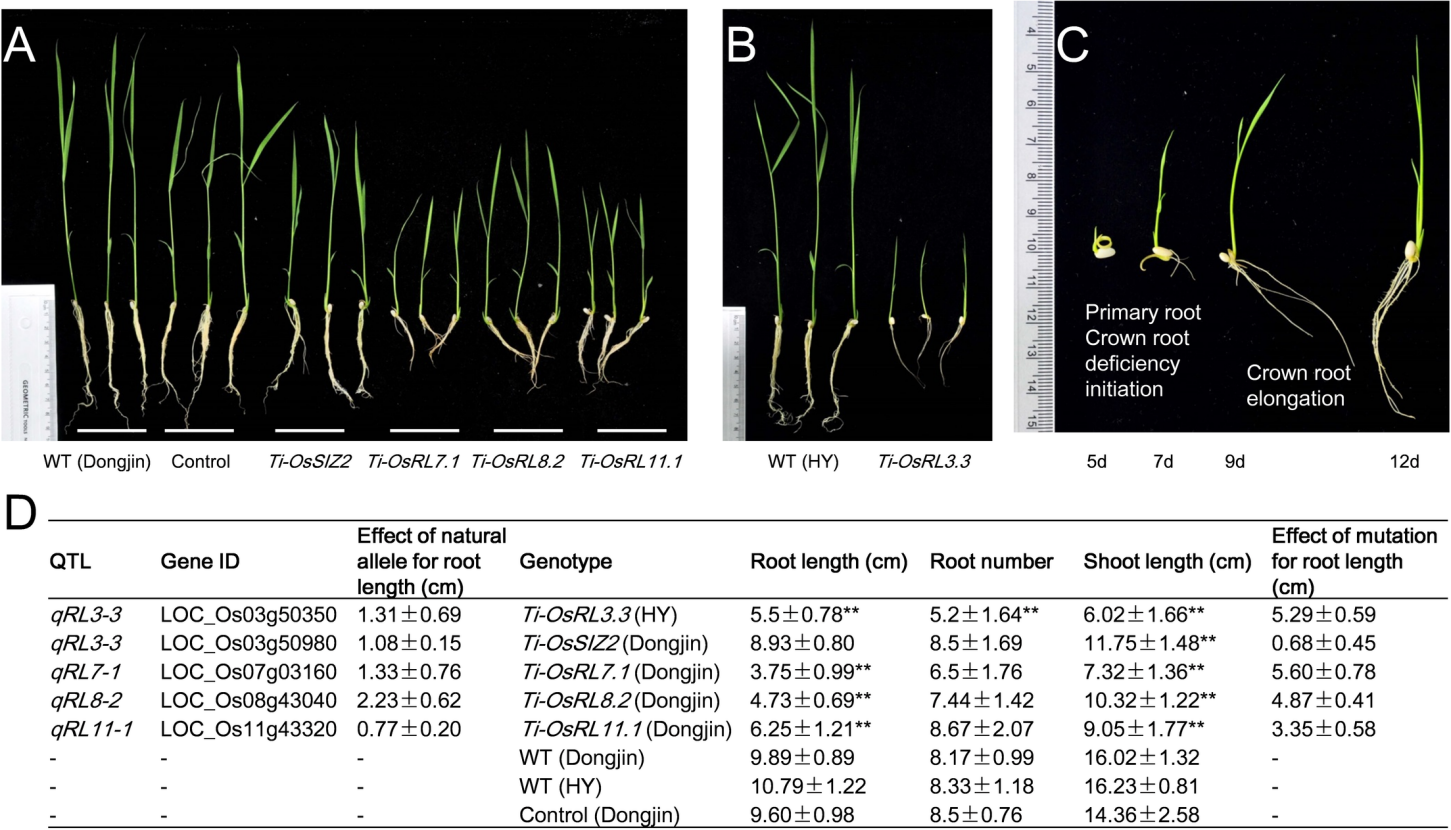
Validation of five QTL genes for root length by T-DNA insertion mutants. (*A*) Comparisons of root lengths of 12-day-old seedlings among the wild type (WT, Dongjin), control (mutant with a T-DNA insertion in *HsfA4a*) and T-DNA insertion mutant lines *Ti-OsSIZ2*, *Ti-OsRL7*.*1*, *Ti-OsRL8*.*2* and *Ti-OsRL11*.*1*. (*B*) Comparisons of root lengths of 12-day-old seedlings among the wild type (WT, Hwayoung) and T-DNA insertion mutant line *Ti-OsRL3*.*3*. (*C*) The root morphologies of line *Ti-OsRL3*.*3* at different growth stages. (*D*) Comparison of root lengths, root numbers and shoot lengths among the wild type, control and different T-DNA insertion lines at 12 days after germination. **, significant (*P* < 0.01) difference between the mutant line compared with WT and control.

To validate the candidate genes related to robust roots, five mutant lines in Dongjin or Hwayoung genetic backgrounds (*Ti-OsRL3*.*3*, *Ti-OsSIZ2*, *Ti-OsRL7*.*1*, *Ti-OsRL8*.*2* and *Ti-OsRL11*.*1*) each containing a T-DNA insertion in one of the five RL candidate genes (LOC_Os03g50350, LOC_Os03g50980, LOC_Os07g03160, LOC_Os08g43040 and LOC_Os11g43320) **(S10 Fig, S16 Table)** for the 4 RL QTLs were evaluated on MS medium for RL, root number and shoot length, together with the Dongjin or Hwayoung wild types and controls (mutant lines in *HsfA4a*, LOC_Os01g54550). The homozygous and heterozygous mutant plants at all loci, except for *Ti-OsSIZ2* showed highly significant reductions in root and shoot growth compared with the wild types and controls **(Fig 6A and 6D)**. *Ti-OsRL3*.*3* mutant (LOC_Os03g50350) plants in particular had no primary root development 5 days after germination, even though the crown roots initiated in the same way as the wild type **(Fig 6B and 6C, S11 Fig)**. Curiously, mutant *Ti-OsSIZ2* (LOC_Os03g50980) showed a slight but non-significant reduction in RL, but a highly significant shoot length reduction when compared with the wild type **(Fig 6D)**. There was a highly significant difference between *Ti-OsRL3*.*3* and the wild type in root number **(Fig 6D)**. *OsSIZ2* was previously shown to complement a dwarf phenotype in Arabidopsis [71], and its homolog (*OsSIZ1*) affected primary root length, crown root number and plant height by regulating auxin accumulation [72,73]. Thus, the detected RL QTL, *qRL3-3*, could be due to either of the two non-synonymous SNPs in LOC_Os03g50350, whereas LOC_Os03g50980 was located in a different LD block and showed no interaction with LOC_Os03g50350 **(S12 Fig)**.

### Pyramiding of robust-root alleles within upland *japonica* in adapting to aerobic conditions

**Fig 7:**
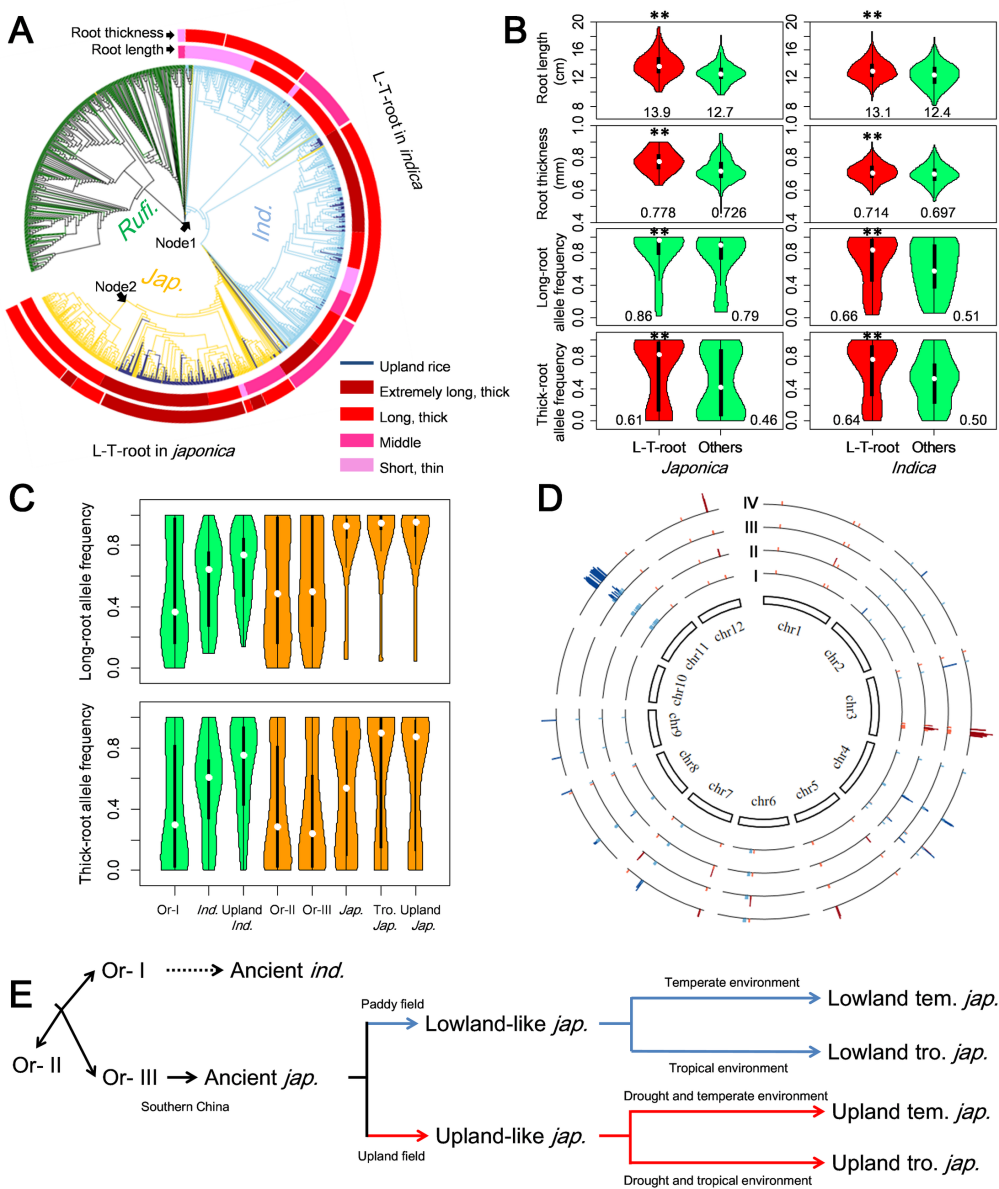
A hypothetical adaptive domestication history of upland rice accessions with robust roots. (*A*) The phylogenetic tree of the 446 *O*. *rufipogon* and 997 *O*. *sativa* accessions based on 5,779 SNPs within the candidate genes for robust roots. (*B*) Comparisons of robust-root phenotypes and allele frequencies between accessions in the long-thick-root (L-T-root) region and other varieties in the corresponding subpopulations; in which the X-axis indicates different groups, and the Y-axis indicates the mean root length and thickness values plotted in the top panel and the long-root and thick-root allele frequencies plotted in the bottom of panel. There are significant differences between red violin and green violin distributions in each plot, and the number represents average value of the adjacent violin. (*C*) Comparisons of frequencies of the long-root alleles and thick-root alleles at candidate genes in *O*. *rufipogon* and *O*. *sativa* subpopulations and ecotypes. Y-axes show allele frequencies for long-roots (upper) and thick-roots (lower), and X-axis represents different groups. Or-I is *indica*-like *O*. *rufipogon* accessions colored blue as *indica*, and Or-II and Or-III are intermediate and *japonica*-like *O*. *rufipogon* accessions colored yellow as *japonica*. (*D*) Ratios of genetic diversity among different groups. The log2-transformed ratios of π_w_/π_J_, π_w_/π_I_, π_non-L-T-root_/π_L-T-root_ in *indica* and π_non-L-T-root_/π_L-T-root_ in *japonica* are plotted against the position on each chromosome, marked by Roman numeralsⅠ,Ⅱ, Ⅲ and Ⅳ. Red and blue represent root length and root thickness candidate genes and the bar lengths indicate log2-transformed ratios. Details are listed in **S17 Table**. (E) Adaptive domestication schematics of upland rice in aerobic conditions. Black lines and dotted line show known and unclear domestication history. Blue and red lines indicate adaptive domestication history of lowland and upland *japonica* ecotypes in tropical and subtropical environments and temperate environment, respectively.

Initially, we constructed a phylogenetic tree of 997 *O*. *sativa* accessions (with 242 upland rice landraces from 21 countries) and 446 wild rice (*O*. *rufipogon*) accessions [2] based on the evenly distributed 90,838 SNPs **(S13 Fig)**. The 242 upland rice landrace accessions were divided into 90 tropical *japonicas*, 41 temperate *japonicas*, 3 *japonicas* (*japonica* but not clearly into tropical or temperate ecotypes), 73 *indicas* and 35 intermediate-types (cultivated rice but not clearly into *japonica* or *indica*). We then constructed a phylogenetic tree of 997 *O*. *sativa* and 446 wild rice accessions **(Fig 7A, S14 Fig)** using 5,779 SNPs in the robust-root candidate genes. The upland rice landraces in *japonica* and *indica* were clearly separated at two nodes after differentiation of *japonica* and *indica*. By further comparing their phenotypic and genotypic differences, we found most upland landraces with robust roots and very high frequencies of long-root and thick-root alleles were clustered in two sub-branches within subspecies *japonica* and *indica*, which were designated L-T-root ecotypes **(Fig 7A and 7B)**. *Japonica* L-T-root ecotypes included 141 upland accessions (89 tropical *japonica*, 36 temperate *japonica*, 1 *japonica* and 15 intermediate), whereas the *indica* L-T-root ecotypes included 59 upland accessions (51 *indica* and 8 intermediate) **(S14 Fig, S1 Table)**. Close examination of the geographical distribution of the upland landraces indicated that the majority of *japonica* L-T-root accessions were from hilly areas with rain-fed farming practices [7,74–78] in South China, Southeast Asia and Africa, as were most *indica* L-T-root landraces from the rain-fed areas of South China, Southeast Asia and India **(S1 Table)**. Clearly, those upland landraces became adapted to the rain-fed upland aerobic conditions of the respective hilly areas. These results suggested that the lower diversity in robust-root candidate genes was a distinctive characteristic of upland rice in adapting to aerobic conditions **(Fig 7A and 7B)**.

We then examined the ancestral states of 59 and 136 non-synonymous SNPs associated with RL and RT; 53 of 59 RL alleles and 108 of 136 RT alleles were detected in the 446 wild accessions. In particular, the frequencies of the long-root and thick-root alleles were lowest in the three wild rice populations, Or-I (49% and 43%, respectively), Or-Ⅱ (53% and 40%) and Or-Ⅲ (55% and 38%) [2], followed by *indica* (57% and 54%), lowland temperate *japonica* (79% and 45%), lowland tropical *japonica* (77% and 58%), and upland temperate *japonica* (85% and 57%), whereas upland tropical *japonica* had the highest long-root and thick-root allele frequencies at 86% and 64% **(Fig 7C)**. The results suggested that almost all alleles were present in wild rice, and there was obvious pyramiding of robust-root alleles in cultivated rice, especially in upland *japonica*.

Given that a reduction in nucleotide diversity was an important selective signature [2], we computed the ratios of genetic diversity between different branches (π_non-L-T-root_/π_L-T-root_) in *japonica* and *indica*. Twenty-six of the 44 RL candidates and 56 of the 97 RT candidates were under selection with greatly reduced diversity in *japonica* L-T-root accessions (π_non-L-T-root_/π_L-T-root_ > 4), whereas only 3 of the 44 RL candidates and 17 of the 97 RT candidates were under selection in *indica* L-T-root accessions **(Fig 7D, S17 Table)**. When comparing the genetic diversity in the wild rice accessions with the *japonica* and *indica* subspecies, (π_w_/π_J_ and π_w_/π_I_), we found 16 of the 44 RL and 9 of 97 RT candidate genes showing strong selective signals in subspecies *japonica*, in contrast to only 1 of 97 RT candidates in *indica* **(Fig 7D, S17 Table)**. The mean value of π_non-L-T-root_/π_L-T-root_ in *japonica* for all candidate genes was 14.6, which is much higher than those of π_non-L-T-root_/π_L-T-root_ in *indica* (2.1), π_w_/π_J_ (3.7) and π_w_/π_I_ (1.2) **(S17 Table)**. Eleven RL candidate genes and 16 RT candidate genes showed very strong selective signals with much higher π_non-L-T-root_/π_L-T-root_ value in *japonica* (> 20), and should be key genes for aerobic adaptation of upland *japonica* **(S17 Table)**. Among them, genes *OsRL3*.*3* and *OsSIZ2* with π_non-L-T-root_/π_L-T-root_ values in *japonica* of 102.1 and 29.6 were shown to be associated with RL by T-DNA insertion mutant lines **(Table 1)**. Further Tajima’s *D* Test was performed for each of the robust-root candidate genes in wild rice, and the four ecotypes of cultivated rice. Tajima’s *D* values of 5, 3, 48, 56 and 92 genes were negative in wild rice, non-L-T-root in *indica*, L-T-root in *indica*, non-L-T-root in *japonica* and L-T-root in *japonica*, respectively. Only one, 0, 7, 4 and 66 gene showed strong selection with Tajima’s *D* < -1 in above separated groups **(S17 Table)**. These observations indicated that significantly more robust-root candidate genes had undergone natural selection during adaptation to aerobic conditions of the upland *japonica* accessions than in other ecotypes. Therefore, we concluded that almost all robust-root alleles were inherited from wild rice, and they gradually accumulated in 4 *japonica* ecotypes during and after domestication of tropical and temperate *japonica* subpopulations, and more robust-root alleles were accumulated by natural and artificial selection in the upland *japonica* ecotype than in other ecotypes in adapting to aerobic conditions **(Fig 7E)**.

## Discussion

### Efficient discovery of moderate- and small-effect alleles for robust roots

Considerable effort has been made to understand the genes underlying variation of complex traits by cloning QTLs. However, QTL cloning by using classical map-based approach is time-consuming and effective only for large-effect QTLs. Cloning QTLs of moderate to small effect is highly challenging owing to the difficulty of phenotyping large populations, particularly for plant root traits such as RL and RT that are difficult to measure under field conditions. In this study we adopted a modified hydroponics system (see Materials and Methods) to measure root traits in rice. This allowed genetic potential to be assessed in the absence of stress [33,79]. Genetically, it remains an unanswered question as to whether large-effect QTLs are primary sources of genetic variation for complex quantitative traits, even though most cloned QTLs in rice and other plants are regulatory genes with large and pleiotropic effects on multiple traits [80,81]. Thus, identification of large numbers of RL and RT QTLs and cloning five of the RL QTLs of small effect in this study should be considered highly successful. This improvement was attributed to our integrated strategy that combined results from high resolution mapping by GWAS and linkage mapping, and comprehensive bioinformatic analyses of genomic, transcriptomic and haplotype data to shortlist QTL candidate genes plus validation of key candidate genes by insertional mutants. With the increasing availability of various kinds of -omics data and genetic stocks such as genome-wide insertional mutants and introgression lines [82–85], it is expected that the integrated strategy demonstrated here will be widely applied in large scale molecular dissection of genes underlying complex traits by efficient cloning and characterization of small- and moderate-effect QTLs. In this respect, our results revealed several unique properties of genes underlying variation of complex traits and shed light on how natural QTL alleles contribute to robust-root systems and adaptation of rice to the aerobic conditions.

Our results indicated that major haplotypes, consisting of non-synonymous SNPs in the coding sequence (CDS) and/or promoter regions within single loci, represent the most important allelic diversity of moderate- and small-effect QTLs underlying robust-root variation in the rice populations. This conclusion was supported by at least five pieces of evidence. First, when examining large numbers of candidate genes in >100 RL and RT QTL regions, we detected 2 to 5 predominant haplotypes (alleles) associated with significant differences in RL or RT, each comprising 2 to 23 non-synonymous SNPs in the CDS or promoter region of each RL and RT QTL candidate locus, including the 5 validated RL QTL genes **(Fig 6, S12 Fig)**. Second, compared with random SNPs, specific haplotypes at single loci showed much greater differentiation in gene frequency among populations, indicating they were important targets of selection during adaptation to aerobic conditions. Third, phenotypic differences between different haplotypes at each of the cloned RL gene loci, though statistically significant, were quite small and did not appear to have pleiotropic effects when compared with lines having insertional mutations in the same genes. Fourth, phenotypic differences between haplotypes at many of these QTL gene loci could be, at least partially, due to differences at the transcriptomic level. This suggests that fine tuning of complex traits in crop improvement can be achieved by careful manipulation of cloned QTL genes at the transcriptional level using various molecular technologies. Fifth, QTL genes affecting the same RL or RT phenotype appeared to have diverse molecular functions, and few of them were regulatory genes **(Table 1)**. These properties distinguish the QTL genes identified in this study from most cloned large-effect QTL genes reported previously. Nevertheless, it remains a challenge to understand how different alleles at moderate- to small-effect QTL loci control complex traits at the molecular level and how they interact with the environment.

### Adaptive domestication of the upland *japonica* ecotype

*O*. *sativa* is well known for its rich diversity and wide subspecies and population differentiation [2,3]. The domestication analysis using 446 *O*. *rufipogon* and 1,083 *O*. *sativa* accessions indicated that *japonica* was first domesticated from a specific population of *O*. *rufipogon* in the Pearl River Valley in southern China [2]. However, adaptation process to aerobic conditions of upland *japonica* rice remains unclear.

Our previous survey of 50,265 rice landraces (33,665 *indica* and 16,784 *japonica*) indicated that upland *japonica* landraces were distributed primarily in hilly areas of four southern and southeastern China provinces (41% in Yunnan, 10% in Guangxi, 16% in Guizhou and 21% in Hainan, respectively) [11] **(S15 Fig)**. The genetic structure of *O*. *sativa* indicated that *japonica* was clearly differentiated between soil water regime ecotypes (uplands and lowlands) in Yunnan [9], Guizhou [86] and many parts of China [4–6], caused by different environments and cropping systems. Based on previous studies of geographical distribution and population structure, we have reasons to believe that upland *japonica* is a unique ecotype with specific morphologies and genotypes associated with drought resistance.

Consistent with previous research, our study verified that robust roots are characteristics of upland rice [10,12,13]. Therefore, we analyzed adaptation process to aerobic conditions of upland *japonica* rice based on 5,779 SNPs in the robust-root candidate genes. The phylogenetic tree showed that there was lower diversity and higher population structure consistency in robust-root candidate genes in the L-T root ecotype in *japonica* (89 tropical *japonica*, 36 temperate *japonica*, 1 *japonica* and 15 intermediate), suggesting that the genotype of robust roots in upland rice was different from that in other ecotypes. By further analysis of nucleotide diversity and allele frequency at robust loci, our study provided new insights into adaptive domestication of the upland rice ecotype. In summary, upland rice accumulated more robust-root alleles in adapting to aerobic conditions during domestication than present in other ecotypes. Additionally, we suggest that the upland *japonica* ecotype is a typical upland rice ecotype with the most robust roots, highest number of robust-root alleles, and strongest selective signals among all tested ecotypes.

## Materials and methods

### Materials

A total of 795 *O*. *sativa* accessions from the 3000 Rice Genome Project (3KRGP) [83,84] were used for identification of RL and RT QTLs, including mini-core collections constructed by Zichao Li in China Agricultural University and 525 lines in the International Rice Molecular Breeding Network constructed by Zhikang Li in Chinese Academy of Agricultural Science [51,52]. All information of cultural type for these rice accessions were from The Catalog of Rice Germplasm Resources in China and IRRI, respectively **(S1 Table)** [11]. For an adaptive domestication analysis, we added 202 additional upland landrace accessions from 19 countries from 3KRGP and 446 *O*. *rufipogon* accessions from Asia and Oceania [2]. The background information of cultural type for the additional 202 upland landrace accessions was from IRRI.

### Phenotyping of root length and root thickness

All 795 *O*. *sativa* accessions were used for phenotyping RL and RT. Two robust-root varieties, HGL and IRAT109, and two lowland temperate *japonica* varieties, Nipponbare and Yuefu, were used as L-T-root and non-L-T-root checks. Four additional long-root varieties (CH1052, CH1086, CH1198 and CH1183) and four non-long-root varieties (CH1179, CH1008, CH1283 and CH1122) were also used as checks in transcriptomic analysis **(S1 Table)**. The hydroponic culture experiment was conducted at China Agricultural University in 2014. Seed of each accession were washed with distilled-water and germinated at 32°C for 64 hours. Five uniformly germinated seeds of each variety were placed into five wells on a plastic foam frame with gauze bottom (30 cm × 42 cm) containing 130 (10 wells × 13 rows) wells, with 110 plants of 22 varieties and 20 plants of two bordering rows **(S16 Fig)**. Two frames were floated in a plastic box (60 cm × 42 cm × 18 cm) containing Yoshida nutrient solution [87]. The pH (5.5) and concentration of the nutrient solution were adjusted twice daily with NaOH and distilled water, and the solution was replaced weekly. Plants were grown under natural conditions but protected with tarpaulins on rainy days. The experiment was conducted twice as two replications from May 24 to June 15 and from June 23 to July 15 in 2014, during which the average daily temperatures fluctuated from 19.9°C to 31.7°C and from 23.9°C to 32.9°C, respectively. Seedlings were allowed to grow for 23 days and then five plants per variety were sampled and measured for RL, RT and root weight using a method described previously [23]. The mean values of all five plants of each accession were used as the input data in the data analyses.

### Genome sequencing

Sequencing data of the 795 and additional upland landraces were obtained from the 3KRGP, and had an average sequencing depth of 15× and generated > 15 million SNPs and > 2 million small InDels when compared with the Nipponbare reference genome [84]. The sequencing data of 446 *O*. *rufipogon* accessions were downloaded from the public database [2] **(S1 File)**.

### Transcriptome sequencing

Six robust-root cultivars and 6 non-robust-root controls were used for transcriptomic analyses. The 12 varieties were planted in pot and field experiments. At the four-leaf stage, roots and shoots of each variety were sampled for extraction of total RNA using TRIzol reagent. High-quality RNA was used for construction of RNA-seq libraries with three biological replicates for each sampled variety. Upon completion of sequencing libraries using the TruSeq RNA sample preparation kit (Illumina), sequencing was performed on an Illumina HiSeq 2500. More than 12.8 million 101-bp simple-ends were generated in each sample. Mapping of RNAseq reads and transcript abundance RPKM was performed by the Cufflinks package version 2.0, based on the rice reference genome and gene model annotation file (GFF transformed GTF file) from the Ensembl database (http://plants.ensembl.org/index.html). The basic information of the transcriptomic data is provided in **S18 Table**.

### Population structure

Based on 3.3 million un-imputed SNPs with missing rates ≤ 50% and minor allele frequencies ≥ 5%, we further extracted 154,516 SNPs with missing rates ≤ 50%, minor allele frequencies ≥ 5% and *r*^2^ of LD ≤ 0.3 using PLINK [88]. The software ADMIXTURE 1.3 was used to calculate varying levels of *K* (*K* = 1–10), and *K* = 8 was a suggesting modeling choice **(S1B and S1C Fig)** [89].

### GWAS and conditional permutation test

Based on 3.3 million un-imputed SNPs with missing rates ≤ 50% and minor allele frequencies ≥ 5%, principal component (PC) and kinship analyses were computed to verify population structure and relationship among the 795 cultivated rice accessions in software GAPIT. The first three PCs were used to construct the PC matrix. We performed GWAS with a Compressed Mixed Linear Model (CMLM) with PC and kinship and GLM with PC using default settings of software GAPIT. A conditional permutation was used to determine the significance threshold. We divided the phenotype (Y) of each accession into the original genotypic effect (G) and the fixed effect of population structure (P). P was estimated by the average effect of each PC in the PC matrix on each individual through regression analysis of each PC on Y; the remainder was G after excluding P from Y. G was randomly reshuffled as Gr, and the new phenotype of each accession was reconstructed as P + Gr. The conditional permutation test was executed using CMLM with the same parameters and PC matrix. A total of 1,000 sets of P + Gr were performed for root length and root thickness. SNPs with -log(*P*) ≥ 2 in the GWAS using original phenotypes were extracted to improve the computational efficiency. Finally a threshold of -log(*P*) = 4 was determined, which was higher than the 95^th^ percentile of 1,000 conditional permutation tests. LD heatmaps of two candidate genes (*OsRL3*.*3* and *OsSIZ2*) were generated using the R package “LD heatmaps”.

### Pooled-χ2-tests

For screening SNPs associated with target traits, we constructed two pools for RL and RT by selecting accessions with extreme phenotypes from typical *indica* and *japonica* populations, then performed a chi-square test on the allele frequency of each SNP executed by in-house Perl scripts. To reduce genetic differences in unrelated target trait between polar pools, we took no account of varieties at PC1 values ranging from -400 to 300. Varieties with PC1 < -400 were defined as typical *japonica* and varieties with PC1 > 300 were considered to be typical *indica* **(Fig 1A)**. Each pool included 20 varieties with extreme phenotypes in typical *indica* and *japonica*, respectively **(S1 Table)**.

### Analysis of T-DNA insertional mutants

After haplotype analysis of candidate genes for robust roots, we obtained T-DNA insertion mutants for 9 RL candidate genes. Six mutant plants containing T-DNA insertions (*Ti-OsRL3*.*3*, *Ti-OsSIZ2*, *Ti-OsRL7*.*1*, *Ti-Os8*.*2*, *Ti-Os11*.*1* and *HsfA4a*) in ‘Dongjin’ or ‘Hwayoung’ background were from the POSTECH Biotech Center, Republic of Korea. Seeds of the six mutant lines were surface-sterilized with 75% ethanol for 2 min and in 20% NaClO for 30 min, and thoroughly washed with sterile water. Medium cultivation experiments were conducted in a glasshouse with a 14h light/10h darkness and temperature 28°C using MS Medium, pH 5.8 **(S17 Fig)**. After 12 days, RL, RT and root weight were measured with a ruler and an electronic scales. DNA was extracted from leaves and was used for identification of homozygous and heterozygous mutants by PCR using gene-specific primers (LP and RP) coupled with a T-DNA-specific primer (RB or LB). PCR was conducted with an initial step of incubation at 95°C for 5 min, followed by a second step of 35 cycles of 95°C for 40s, 58°C (55°C) for 40s, and 72°C for 1 min 20s. The PCR products were genotyped by a sequencing company.

### Phylogenetic analysis

We downloaded the SNP set (7,970,357) of 1,529 rice lines from a recent study and selected SNP data for the 446 wild rice accessions that were simply classified into three types Or-I, Or-II and Or-III in this study [2]. Since this dataset used IRGSP4 Nipponbare genome as reference, we extracted the 200 bp flanking regions from each side of the target SNP (200+1+200 = 401 bp) from the IRGSP4 reference and mapped 401 bp sequences to the RGAP 7 reference by BWA software [90]. We converted the SNP coordinate of IRGSP4 to RGAP 7 based on the mapping result and then integrated the genetic information for 997 cultivated rice accessions and 446 wild rice accessions following extraction of 5,779 SNPs within robust-root candidate genes (44 genes for RL and 97 genes for RT) from 1,443 accessions **(S1 File)**. A neighbor-joining tree based on the polymorphisms was generated using Tassel 5 and Mega 6, and the nucleotide diversity (π) [91] was calculated using an in-house Perl script **(S1 File)**. We also extracted 90,838 SNPs evenly distributed throughout the genomes of 1,443 accessions, and a constructed neighbor-joining tree as control.

## Supporting information

S1 Fig Quality test of genome sequencing, and the population structure and kinship of 795 *O*. *sativa* (rice) accessions. (DOCX)

S2 Fig Histograms of phenotypic diversity in root traits of the whole population and different ecotypes of rice. (DOCX)

S3 Fig Distribution of root weight among different rice ecotypes. (DOCX)

S4 Fig Genome-wide association study of root length under GLM. (DOCX)

S5 Fig Genome-wide association study of root thickness under GLM. (DOCX)

S6 Fig The conditional permutation test for determining the threshold in the whole population using the CMLM model. (DOCX)

S7 Fig Identification of putative functional genes in 19 QTL regions detected by GWAS. (DOCX)

S8 Fig Haplotype analyses of 4 key root length candidate genes. (DOCX)

S9 Fig Haplotype analyses of 14 key root thickness candidate genes. (DOCX)

S10 Fig Genotypic analysis of four mutant lines and control plants. (DOCX)

S11 Fig Phenotypes of primary roots in wild type Dongjin and T-DNA insertion mutant *Ti-OsRL3*.*3* lines. (DOCX)

S12 Fig LD heatmap for 9 non-synonymous SNPs in *OsRL3*.*3* and *OsSIZ2* for *qRL3-3*. (DOCX)

S13 Fig The neighbor-joining tree of 997 *O*. *sativa* and 446 wild *O*. *rufipogon* accessions based on evenly distributed 90,838 SNPs across the genome. (DOCX)

S14 Fig Phylogenetic relationships among the sampled population based on SNPs within the robust-root candidate genes. (DOCX)

S15 Fig Geographical distributions of 3,027 upland *japonica* landraces from 50,265 rice landraces in China. (DOCX)

S16 Fig The phenotyping system for evaluating rice root traits under the hydroponic conditions. (DOCX)

S17 Fig Root phenotypes of wild type Dongjin, control (mutant with T-DNA insertion in known gene *HsfA4a*), and T-DNA insertion mutant lines *Ti-OsSIZ2*, *Ti-OsRL7*.*1*, *Ti-OsRL8*.*2* and *Ti-OsRL11*.*1*. (DOCX)

S1 Table Description of 795 rice accessions, 202 additional landraces and 446 wild rice accessions. (XLSX)

S2 Table Descriptive statistics of 3 root traits among different ecotypes. (XLSX)

S3 Table Summary of QTLs and SNPs associated with root length detected by GWAS. (XLSX)

S4 Table Summary of QTLs and SNPs associated with root thickness detected by GWAS. (XLSX)

S5 Table Distribution of different allele types at 857 SNP sites in pools of long and short roots and corresponding subpopulations. (XLSX)

S6 Table Long-root allele frequencies of 230 SNP sites with significant difference between pools of long and short roots. (XLSX)

S7 Table Description of common root length and root thickness QTLs from GWAS and linkage mapping in rice. (XLSX)

S8 Table Summary of 119 SNPs from 7 root length common QTL identified by GWAS and linkage mapping. (XLSX)

S9 Table Summary of 146 SNPs in 81 genes identified by GWAS, linkage mapping and pooled-χ2-tests. (XLSX)

S10 Table Screening of SNPs related to root thickness in GWAS-QTLs by pool-χ2-test and linkage mapping. (XLSX)

S11 Table RPKM of 44 root length candidate genes in long-root varieties and controls. (XLSX)

S12 Table SNPs leading amino acid polymorphism for 44 root length candidate genes in pools, parents and populations. (XLSX)

S13 Table InDels leading amino acid polymorphism for 20 root length candidate genes in pools and populations. (XLSX)

S14 Table Analysis of expression and amino acid variation for 97 root thickness key candidate genes. (XLSX)

S15 Table SNPs leading amino acid polymorphisms for root thickness candidate genes in pools, parents and populations. (XLSX)

S16 Table Primers used in this study. (XLSX)

S17 Table Ratio of genetic diversity and Tajima’s *D* test of root length and root thickness candidate genes among different populations. (XLSX)

S18 Table RPKM of all annotated gene in long-root varieties and controls. (XLSX)

S1 File Main Perl scripts used in our study. (ZIP)
