# Supplementary material for: Loci and natural alleles underlying robust roots and adaptive domestication of upland ecotype rice in aerobic conditions
Source: PLoS Genet. 2018 Aug 10;14(8):e1007521. doi: 10.1371/journal.pgen.1007521 (PMC6086435; doi:10.1371/journal.pgen.1007521)
Supplement: S1 Fig — (DOCX) [file pgen.1007521.s001.docx]

**Fig S1.** Quality test of genome sequencing, and the population structure and kinship of 795 *O. sativa* (rice) accessions. (*A*) The missing data rates of all 15,133,187 SNPs detected in our population. (*B*) Genetic structure of the panel analyzed using 154,516 SNPs with missing rates ≤ 50%, minor allele frequencies ≥ 5% and *r*^2^ of linkage disequilibrium (LD) ≤ 0.3. (*C*) Cross-validation plot. (*D*) The Kinship matrix. Kinship was performed using 3.3 million SNPs with missing data rates ≤ 50% and minor allele frequencies ≥ 5%.
