# Supplementary material for: Loci and natural alleles underlying robust roots and adaptive domestication of upland ecotype rice in aerobic conditions
Source: PLoS Genet. 2018 Aug 10;14(8):e1007521. doi: 10.1371/journal.pgen.1007521 (PMC6086435; doi:10.1371/journal.pgen.1007521)
Supplement: S2 Fig — (DOCX) [file pgen.1007521.s002.docx]

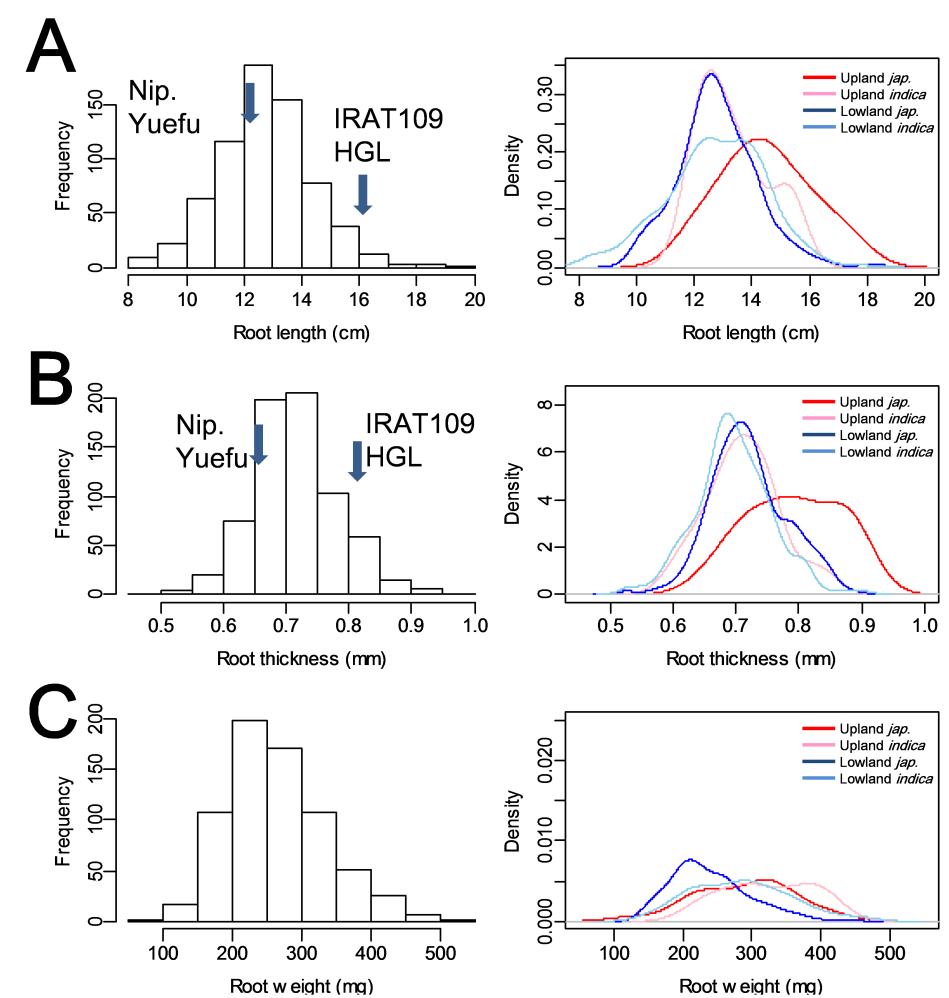


**Fig S2.** Histograms of phenotypic diversity in root traits of the whole population and different ecotypes of rice. (*A*) Root length, (*B*) root thickness and (*C*) root weight for the whole population (left), and frequency distributions among different ecotypes (right). Blue arrows show phenotypic values of the 4 varieties used as materials for later transcriptome analysis.
