# Supplementary material for: Loci and natural alleles underlying robust roots and adaptive domestication of upland ecotype rice in aerobic conditions
Source: PLoS Genet. 2018 Aug 10;14(8):e1007521. doi: 10.1371/journal.pgen.1007521 (PMC6086435; doi:10.1371/journal.pgen.1007521)
Supplement: S3 Fig — (DOCX) [file pgen.1007521.s003.docx]

**Fig S3.** Distribution of root weight among different rice ecotypes. The Y-axes show the median (white point), 95% of confidence intervals (black bars) and range (colored shapes) of the trait phenotypes. The X-axes indicate different ecotypes, ordered by upland *japonica*, upland *indica*, lowland *japonica* and lowland *indica*. Numbers above violins are mean phenotypic values of different populations. Ecotypes colored red with * were significantly higher than those colored green (*p* < 0.05 detected by one-way ANOVA).
