# Supplementary material for: Loci and natural alleles underlying robust roots and adaptive domestication of upland ecotype rice in aerobic conditions
Source: PLoS Genet. 2018 Aug 10;14(8):e1007521. doi: 10.1371/journal.pgen.1007521 (PMC6086435; doi:10.1371/journal.pgen.1007521)
Supplement: S4 Fig — (DOCX) [file pgen.1007521.s004.docx]

**Fig S4.** Genome-wide association study of root length under GLM. The Manhattan plots (left) and quantile-quantile (Q-Q) plots (right) for root length in (*A*) whole population, (*B*) *japonica* subpopulation, and (*C*) *indica* subpopulation. For Q-Q plot, the X- and Y-axes show -log10 transformed expected *P* values and -log10 transformed observed *P* values.
