# Supplementary material for: Loci and natural alleles underlying robust roots and adaptive domestication of upland ecotype rice in aerobic conditions
Source: PLoS Genet. 2018 Aug 10;14(8):e1007521. doi: 10.1371/journal.pgen.1007521 (PMC6086435; doi:10.1371/journal.pgen.1007521)
Supplement: S6 Fig — (DOCX) [file pgen.1007521.s006.docx]

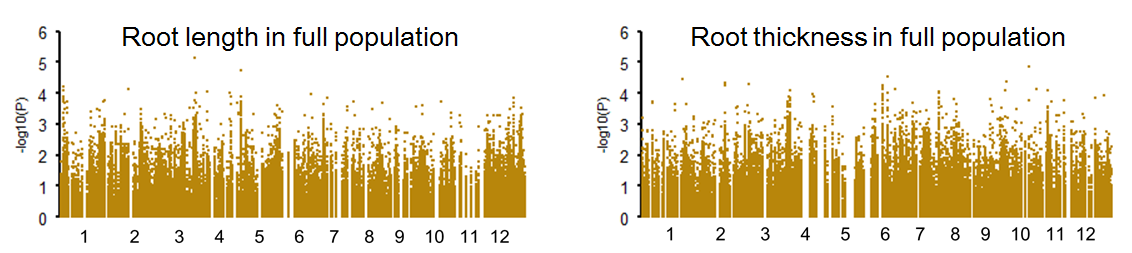


**Fig S6.** The conditional permutation test for determining the threshold in the whole population using the CMLM model.
