# Supplementary material for: Loci and natural alleles underlying robust roots and adaptive domestication of upland ecotype rice in aerobic conditions
Source: PLoS Genet. 2018 Aug 10;14(8):e1007521. doi: 10.1371/journal.pgen.1007521 (PMC6086435; doi:10.1371/journal.pgen.1007521)
Supplement: S7 Fig — (DOCX) [file pgen.1007521.s007.docx]

**Fig S7.** Identification of putative functional genes in 19 QTL regions detected by GWAS. (*A*) *qRL1-1*. (*B*) *qRL1-2*. (*C*) *qRL1-3*. (*D*) *qRL2-1*. (*E*) *qRL3-1*. (*F*) *qRL3-2*. (*G*) *qRL3-3*. (*H*) *qRL4-1*. (*I*) *qRL4-2*. (*J*) *qRL5-1*. (*K*) *qRL5-2*. (*L*) *qRL6-1*. (*M*) *qRL7-1*. (*N*) *qRL7-2*. (*O*) *qRL8-1*. (*P*) *qRL9-1*. (*Q*) *qRL10-1*. (*R*) *qRL12-1*. (*S*) *qRL12-2*. Top of each panel shows the entire region of each QTL detected by GWAS using the whole population and CMLM, in which X-axes indicate the physical positions (Mb), and negative log_10_-transformed *P* values are plotted on the Y-axes. Points show positions and -log(*P*) values of all SNPs in the QTL region. Among them, red points indicate SNPs with significant differences of allele frequency between the long-root and short-root pools and those verified by linkage mapping of bi-parental populations; pink points represent SNPs showing significant differences in allele frequency between pools for QTLs detected only by GWAS; and skyblue points show SNPs except of red and pink points. In bottom of each panel, annotated genes are indicated by skyblue boxes, and candidate genes are indicated by red boxes.
