# Supplementary material for: Loci and natural alleles underlying robust roots and adaptive domestication of upland ecotype rice in aerobic conditions
Source: PLoS Genet. 2018 Aug 10;14(8):e1007521. doi: 10.1371/journal.pgen.1007521 (PMC6086435; doi:10.1371/journal.pgen.1007521)
Supplement: S8 Fig — (DOCX) [file pgen.1007521.s008.docx]

**Fig S8.** Haplotype analyses of 4 key root length candidate genes. In the gene structure plots (left), positions colored red show differential loci among haplotypes responsible for the observed significant differences between or among different haplotypes in root length. Among root length violin maps of different haplotypes in subpopulations *japonica* (upper right) and *indica* (lower right), different letters above the violins indicate significant differences (*p* < 0.05) when analyzed by Duncan’s test or Independent-sample T-tests.
