# Supplementary material for: Loci and natural alleles underlying robust roots and adaptive domestication of upland ecotype rice in aerobic conditions
Source: PLoS Genet. 2018 Aug 10;14(8):e1007521. doi: 10.1371/journal.pgen.1007521 (PMC6086435; doi:10.1371/journal.pgen.1007521)
Supplement: S9 Fig — (DOCX) [file pgen.1007521.s009.docx]

**Fig S9.** Haplotype analyses of 14 key root thickness candidate genes. In the gene structure plots (left), positions colored red show differential loci among haplotypes with significant root thickness differences. Among root thickness violin maps of different haplotypes in subpopulations *japonica* (upper right) and *indica* (lower right), different letters above the violins indicate significant differences (*p* < 0.05) when analyzed by Duncan’s test or Independent-sample T-tests. "F", "J" and "I" in bracket showed that the GWAS QTL was detected in whole population, *japonica* and *indica*, respectively.
