# Supplementary material for: Loci and natural alleles underlying robust roots and adaptive domestication of upland ecotype rice in aerobic conditions
Source: PLoS Genet. 2018 Aug 10;14(8):e1007521. doi: 10.1371/journal.pgen.1007521 (PMC6086435; doi:10.1371/journal.pgen.1007521)
Supplement: S10 Fig — (DOCX) [file pgen.1007521.s010.docx]

**Fig S10.** Genotypic analysis of four mutant lines and control plants. (*A*) Schematic representation of the control (*HsfA4a,* LOC_Os01g54550), *Ti-OsSIZ2* (*OsSIZ2*, LOC_Os03g50980), *Ti-OsRL7.1* (*OsRL7.1*, LOC_Os07g03160), *Ti-OsRL8.2* (*OsRL8.2*, LOC_Os08g43040) and *Ti-OsRL11.1* (*OsRL11.1*, LOC_Os11g43320); (*B*) Genotyping of the control plants and four mutant lines (the primer sets used in PCR are included in **Table S16**.
