# Supplementary material for: Loci and natural alleles underlying robust roots and adaptive domestication of upland ecotype rice in aerobic conditions
Source: PLoS Genet. 2018 Aug 10;14(8):e1007521. doi: 10.1371/journal.pgen.1007521 (PMC6086435; doi:10.1371/journal.pgen.1007521)
Supplement: S12 Fig — (DOCX) [file pgen.1007521.s012.docx]

**Fig S12.** LD heatmap for 9 non-synonymous SNPs in *OsRL3.3* and *OsSIZ2* for *qRL3-3*. Block box shows region with weak local LD (*r*^2^ < 0.4).
