# Supplementary material for: Loci and natural alleles underlying robust roots and adaptive domestication of upland ecotype rice in aerobic conditions
Source: PLoS Genet. 2018 Aug 10;14(8):e1007521. doi: 10.1371/journal.pgen.1007521 (PMC6086435; doi:10.1371/journal.pgen.1007521)
Supplement: S13 Fig — (DOCX) [file pgen.1007521.s013.docx]

**Fig S13.** The neighbor-joining tree of 997 *O. sativa* and 446 wild *O. rufipogon* accessions based on evenly distributed 90,838 SNPs across the genome. *O. rufipogon*, *japonica*, and *indica* accessions are colored green, blue and cyan. Tropical upland *japonica*, temperate upland *japonica*, upland *japonica* (*japonica* but not clearly into tropical or temperate ecotypes), upland *indica* and intermediate upland types are marked by red, pink, gray, blue and black dots.
