# Supplementary material for: Loci and natural alleles underlying robust roots and adaptive domestication of upland ecotype rice in aerobic conditions
Source: PLoS Genet. 2018 Aug 10;14(8):e1007521. doi: 10.1371/journal.pgen.1007521 (PMC6086435; doi:10.1371/journal.pgen.1007521)
Supplement: S14 Fig — (DOCX) [file pgen.1007521.s014.docx]

**Fig S14.** Phylogenetic relationships among the sampled population based on SNPs within the robust-root candidate genes. (*A*) The neighbor-joining tree for 997 *O. sativa* accessions and 446 *O. rufipogon*. The *O. rufipogon*, non-L-T-root in *japonica*, L-T-root in *japonica*, non-L-T-root in *indica* and L-T-root in *indica* are colored in green, blue, pink, cyan, darkgreen, respectively. Tropical upland *japonica*, temperate upland *japonica*, upland *japonica* (*japonica* but not clearly into tropical or temperate ecotypes), upland *indica* and intermediate upland types are marked by red, pink, gray, blue and black dots. (*B*) The neighbor-joining tree of 186 accessions in the L-T-root region of *japonica*.
