# Supplementary material for: Loci and natural alleles underlying robust roots and adaptive domestication of upland ecotype rice in aerobic conditions
Source: PLoS Genet. 2018 Aug 10;14(8):e1007521. doi: 10.1371/journal.pgen.1007521 (PMC6086435; doi:10.1371/journal.pgen.1007521)
Supplement: S16 Fig — (DOCX) [file pgen.1007521.s016.docx]

**Fig S16.** The phenotyping system for evaluating rice root traits under the hydroponic conditions. (*A*) Rice seedlings in the hydroponic system; (*B*) Root phenotypes of rice seedlings and (*C*) the phenotypic diversity in root length.
