# Supplementary material for: Loci and natural alleles underlying robust roots and adaptive domestication of upland ecotype rice in aerobic conditions
Source: PLoS Genet. 2018 Aug 10;14(8):e1007521. doi: 10.1371/journal.pgen.1007521 (PMC6086435; doi:10.1371/journal.pgen.1007521)
Supplement: S17 Fig — (DOCX) [file pgen.1007521.s017.docx]

**Fig S17.** Root phenotypes of wild type Dongjin, control (mutant with T-DNA insertion in known gene *HsfA4a*), and T-DNA insertion mutant lines *Ti-OsSIZ2*, *Ti-OsRL7.1*, *Ti-OsRL8.2* and *Ti-OsRL11.1*. (A) Planting diagram for root length phenotyping; Comparisons of root and shoot growth among wild type, control and mutant lines at (B) 6 and (C) 12 days post germination.
